# Revisiting Many-Body Interaction Heat Current and Thermal Conductivity Calculations Using the Moment Tensor Potential/LAMMPS Interface

**DOI:** 10.1021/acs.jctc.4c01659

**Published:** 2025-03-29

**Authors:** Siu Ting Tai, Chen Wang, Ruihuan Cheng, Yue Chen

**Affiliations:** † Department of Mechanical Engineering, The University of Hong Kong, Pokfulam Road, Hong Kong SAR, China; ‡ Institute for Advanced Study, Shenzhen University, Shenzhen 518060, China

## Abstract

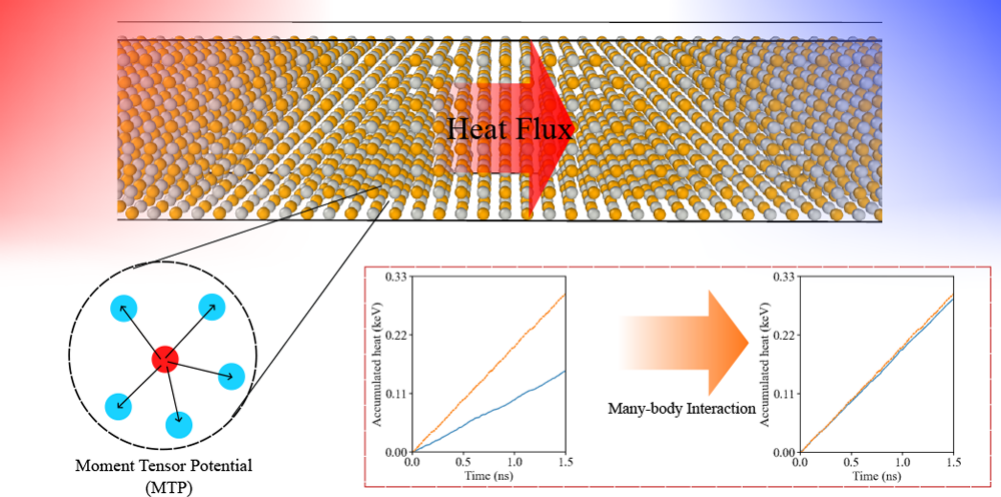

The definition of heat current operator for systems for nonpairwise additive interactions and its impact on related lattice thermal conductivity (κ_*L*_) via molecular dynamics (MD) simulation are ambiguous and controversial when migrating from empirical potential models to machine learning potential (MLP) models. Herein, we study and compare the significance of many-body interaction with heat current computation in one of the most popular MLP models, the moment tensor potential (MTP). Nonequilibrium MD simulations and equilibrium MD simulations among four different materials were performed, and inconsistencies in energy conservation between the simulation thermostat and the pairwise calculator were found. A new virial stress tensor expression with a many-body heat current description was integrated inside the MTP, and we uncovered the influence of the modification that could alter the κ_*L*_ results by 29–64% using the equilibrium MD computational approach. Our work demonstrates the importance of a many-body description during thermal analysis in MD simulations when MLPs are of concern.

## 1 Introduction

MLP has recently gained popularity in the application of computational materials engineering, especially for molecular dynamics (MD) simulations. Before the introduction of MLP, empirical potential functions, such as the Born–Mayer–Huggins potential,^1^ the Tersoff^2^ potential, and the Sillinger and Weber potential^3^ are implemented under a limited description to the interatomic interaction when used in conventional MD. They often fail to describe interactions of defects, surfaces, or metastable states. MLP alternatively provides a more generalized, data-driven construction of the interatomic interaction model. Various established MLP models, for instance, the neuroevolution potential (NEP),^4^ the Gaussian approximation potential (GAP),^5^ or the MTP,^6^ enable machine learning and training of the mathematics models coefficient over an expandable basis function set with desirable accuracy to encompass the complexity of many-body interaction within the local atomic environment neighborhood. MLP empowers more versatile applications such as simulations of the hydrogenation of amorphous silicon using GAP^7^ and interfacial diffusion between the Ge–Se alloy and Ti metal using MTP^8^ that are not possible to accurately describe with the conventional oversimplified potential due to complex physical interactions. MLPs are now crucial in the simulation field for material researchers to investigate novel materials and explore advanced applications.

A readily available software package, machine learning interatomic potential (MLIP), has been developed by Novikov et al.^6^ to address the need for a robust and computationally effective MLP. The MLIP features the MTP machine learning model with an interface to the software LAMMPS.^9^ Studies have demonstrated the package as an effective interatomic potential in the research of thermal transport and phonon properties of different materials such as the superionic conductor AgCrSe_2_,^10^ wurtzite boron arsenide,^11^ and multiple 2D materials.^12^ Compared with other MLP models, MTP and its software package deliver an easy-to-use toolkit with better balance among performance, speed, and accuracy as compared with GAP, spectral neighbor analysis potential, and neural network potential.^13^ These advantages of the MLIP program introduce new opportunities to the material research community to predict lattice thermal conductivity (κ_L_) and the thermal transport mechanism of a wide range of materials through MD simulation.

The study of the thermal conductivity of materials is a crucial application of molecular simulation analysis in which MLPs are actively employed within the field. Experimental measurements often show challenges in determining heat transport properties of materials due to limitations in sample preparation and accuracy of the empirical model. Theoretical computation results of κ_L_ can be approximated through MD simulation approaches in which uncertainty is controlled. Popular MD techniques, namely, equilibrium MD (EMD),^14^ nonequilibrium MD (NEMD),^15^ reverse nonequilibrium MD (RNEMD),^16^ and approach-to-equilibrium MD (AEMD),^17^ have been generally employed by researchers. The EMD based on the Green–Kubo formula facilitates the computation of κ_*L*_ by relating heat current autocorrelation functions under the dissipation–fluctuation theorem as follows:^14^
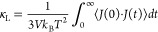
1where κ_L_, *V*, *k*_B_, *T*, and *J* represent the thermal conductivity coefficient, volume of simulation cells, Boltzmann’s constant, temperature, and the heat current, respectively.

The NEMD or direct method^15^ is conducted using a simulated, steady temperature gradient to study the heat current and κ_L_ using Fourier’s law for heat conduction, making it directly analogous to an experimental measurement. A heat source and a heat sink sandwiched with a microcanonical *NVE* ensemble domain are usually defined to provide steady heat current conditions. The third method, the RNEMD approach, reverses the conventional paradigm in NEMD heat transport simulation by imposing a predetermined heat flux across the simulation domain instead of enforcing a temperature gradient on it. This technique suggested a faster convergence time requirement and mitigated the necessity of heat current evaluation. The last method, the approach-to-equilibrium technique,^17^ aims at simulating and capturing the out-of-equilibrium system responses of the temperature gradient when it returns to the equilibrium state. Two connecting domains, equilibrated at different temperatures, are modeled in a controlled simulation. The MD then allows energy flows between the two temperature blocks under a microcanonical *NVE* simulation. By fitting the evolution to the temperature difference between the two blocks Δ*T* with the first exponential decay time τ, the thermal conductivity of a material can be estimated.

Although both *EMD* and *NEMD* are considered more popular in analyzing the κ_*L*_ of various material systems, they rely on the evaluation of the heat current within the model, which *RNEMD* and *AEMD* do not. There exist plenty of reports and uses of MTP in determining κ_*L*_ using MD simulation approaches.^18^ However, there is a lack of focus on the computation of the heat current itself. Given the popularity of *EMD* and direct approaches, as well as their fundamental principles for resolving the heat current component of the system when obtaining κ_*L*_, it is crucial to understand how the MTP model evaluates the heat flux component of the system and address its impact on the two common simulation methodologies, *EMD* and *NEMD*.

The heat current evaluation for MTP was implemented within the LAMMPS/MLIP interface, but its derivation is ambiguous. The essence of the heat current calculation falls into Hardy’s expression of heat current in a solid.^19^
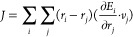
2where *r*_*i*_, *v*_*i*_, and *E*_*i*_ represent position, velocity, and total energy of atom *i*, respectively. This expression can be further simplified and has been expressed as eq 3 in the LAMMPS package,^9,20^ where *e*_*i*_ is the per-atom energy and *W*_*i*_ is the atomic virial stress tensor.
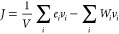
3In the MLIP packages, the potential component of the heat current, presented as the second summation term on the right-hand side in eq 3, was calculated as
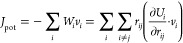
4

Recently, a modification to the LAMMPS MD package brought new insight into how to handle and compute heat current in a simulation problem. Boone et al.^20^ and Surblys et al.^21^ suggested including the many-body interaction in the atomic stress formula, replacing pairwise virial stress with a centroid virial stress tensor. The modification results in a higher heat current value in their example of butane, octane, and polystyrene, which led to the highest reduction of up to 25% κ in butane. To transfer the idea of many-body heat current computation into MLP models, a generalized formula was developed,^22^ which is limited to few MLP systems, such as NEP,^4^ and the SchNet message-passing neural network semilocal MLP.^23^ The potential contribution of the heat current can be written as eq 5
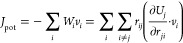
5

It is worth noting that the expression for the atomic virial stress of atom *i* is different with a subscript index of the atomic energy derivative term that swaps from *i* to *j* when comparing eq 4 with eq 5. The derivation of the many-body heat current formula suggested that the original heat current expressed by the MLIP was a pairwise interaction expression that assumes a premise of *U*_*i*_ = *U*_*j*_, where energy between a pair of atoms only depends on their distance *r*_*ij*_ as discussed in ref (22). Although the MTP potential provides a mathematical model that considers the many-body interactions between the atomic local environment when evaluating the energy and forces of the system, the MLIP software construction shows a lack of ability to express the heat current under a many-body context. We intend to demonstrate the significance of employing the generalized many-body heat current formula with examples of various materials in this paper.

## 2 Computation Details for MD Simulations

With our aim to illustrate the needs and effect of modifying the heat current description to express the nonpairwise contribution in MTP, we perform four sets of MD simulations over different materials, namely, PbTe, amorphous Sc_0.2_Sb_2_Te_3_, graphene, and BAs. These examples were selected to cover a range of orders of magnitude from 10^–1^ to 10^3^ W/mK in κ_*L*_ and a variety of spatial complexities and symmetry orders. The four chosen materials are able to generally validate the potential impact of the generalized many-body heat current formula on the computed heat current and its influence on κ_*L*_. Both *NEMD* and *EMD* were performed for each of the mentioned material examples to demonstrate the significance of the heat current formulation modification in this work.

MTP interatomic potentials for the four respective materials are prepared by passive MTP training utilizing the MLIP packages. Training sets for PbTe, amorphous Sc_0.2_Sb_2_Te_3_, and graphene are extracted from established works of Cheng et al.,^24^ Wang et al.,^25^ and Rowe et al.,^26^ respectively. The training set for BAs was prepared independently in a similar manner described in Mortazavi’s work^27^ through sampling atomic structure configurations in ab initio MD simulation trajectories of a 5 × 5 × 5 supercell of BAs using the Vienna ab initio simulation package (VASP).^28^ Forces and energies of 408 configurations sampled from 200 to 900 K were evaluated under the density functional theory (DFT) framework using the VASP package. The exchange-correlation functional was approximated using the projector-augmented wave method^29^ incorporated with the Perdew–Burke–Ernzerhof generalized gradient approximation. A 2 × 2 × 2 Γ-centered *k*-point mesh with a kinetic energy cutoff of 600 eV was employed. The energy convergence threshold was set to be 10^–7^ eV for all of the single-point self-consistent energy calculations.

**Figure 1:**
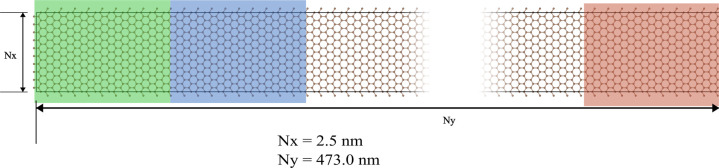
Simplified single-layered graphene model for the *NEMD* simulation. Green, blue, and red sections represent the fixed end, the heat sink, and the heat source, respectively.

The *NEMD* simulation was employed to calculate the room-temperature heat current for PbTe, amorphous Sc_0.2_Sb_2_Te_3_, graphene, and BAs using the MTP potentials trained. We adopted a similar heat current validation approach in ref (30) to uncover the necessity of expressing the heat current through the many-body heat current formula when incorporating the MTP potential. A steady temperature gradient centered at 300 K along the *y*-direction of the periodic orthogonal models was employed with a fixed end to insulate heat flow between the heat sink and the source. Figure 1 shows an example of the schematic diagram of a single-layered graphene model of 44,400 atoms. The model spans a length of around 473 nm, a width of 2.5 nm, and a 3 nm vacuum space in the out-of-plane direction, which we found to be a decent balance between the simulation dimension with at least 300 nm in the characteristic heat flow direction and a computational expense of around 10000 CPU hours for each simulation.

The other two bulk material models of PbTe and amorphous Sc_0.2_Sb_2_Te_3_ were structured in similar configurations with reduced cell sizes since their expected thermal conductivities are significantly lower with a shorter phonon mean free path compared to graphene. The lengths of the simulation cells were chosen to be 32 and 45.7 nm, respectively. The amorphous Sc_0.2_Sb_2_Te_3_ model was built to contain a much higher number of atoms of 18,000 due to the larger unit cell for representing a disordered structure. The model of BAs was scaled to have a length of 486 nm, which is similar to the graphene structure as their room-temperature phonon mean free path is comparable at the range of 2–10 μm.^31,32^

All four sets of *NEMD* simulations were carried out in two stages. A time step of 1 fs was chosen for all four models. The simulation domain, except for the fixed end section, was first equilibrated at a center temperature of 300 K for 200 ps under an *NVT* canonical ensemble. The system was then run for another 2 ns under an *NVE* ensemble with a steady and uniform temperature gradient applied to the heat source and heat sink through a Langevin thermostat at 350 and 250 K, respectively. We considered the first 0.5 ns of the run to stabilize the system under such a temperature gradient and recorded the heat current accounted for by the Langevin heat baths and the calculated heat current through the MLIP interface for the remaining 1.5 ns of the run.

Another angle to visualize the impact of using the many-body heat current formula in the MTP potential is the change in κ_*L*_ computed by *EMD* methods. This technique involves intensive evaluation of heat current using the MLIP interfaces and brings insight into its legitimacy. The numbers of atoms in each model were selected to be 1728, 11520, 1800, and 1728 for PbTe, amorphous Sc_0.2_Sb_2_Te_3_, graphene, and BAs, respectively. Five independent simulations, each containing 10 complete autocorrelation windows, were performed on each material. The first four correlation windows were discarded, and 30 sampled heat current autocorrelation functions were completed for all four examples. The correlation times were chosen to be 40 and 80 ps for PbTe and amorphous Sc_0.2_Sb_2_Te_3_, respectively. Graphene and BAs have a higher κ_*L*_ and use a correlation time of 1000 ps. The simulation time step was set to be 1 fs. All simulations were first equilibrated at 300 K under an *NVT* ensemble and later ran a length covering 10 correlation time windows under the *NVE* ensemble. The heat current autocorrelation function was calculated in the latter half of the MD simulations as implemented within the LAMMPS package, and we computed the κ_*L*_ of each material subjected to both the original and the modified heat current formula using eq 1.

## 3 Results and Discussion

### 3.1 *NEMD* Simulation for Direct Heat Current Evaluation

**Figure 2:**
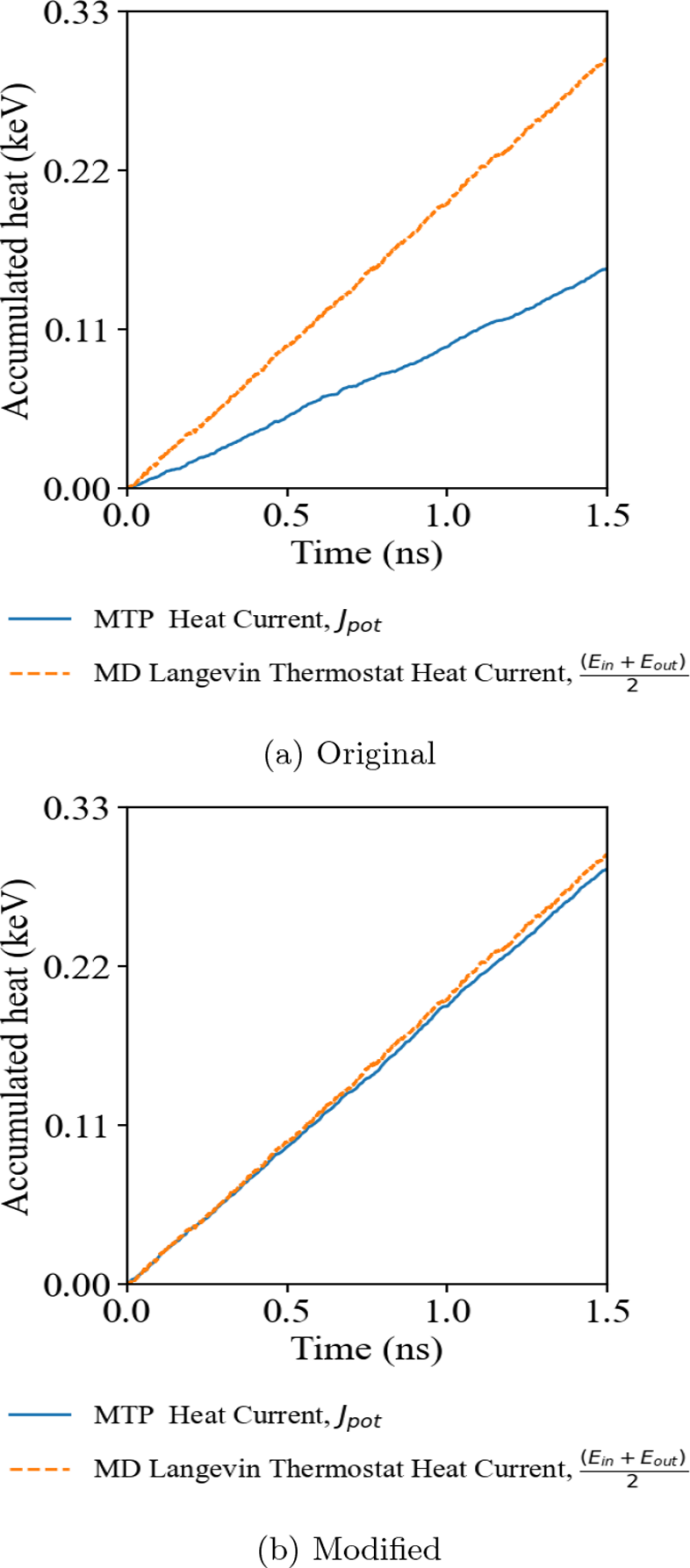
Accumulative heat of *NEMD* simulations of PbTe using (a) the original MLIP interface and (b) the modified MLIP interface with the many-body heat current formula correction.

We first consider the cubic PbTe crystal. The *NEMD* simulation result in Figure 2a illustrates the overall cumulative heat passed through the heat source and sink as an orange dashed line, which was evaluated within the LAMMPS implementation of the ensemble thermostat. On the other hand, the cumulative heat evaluated by MTP across the simulation domain, drawn in the blue line, deviates from the overall *NEMD* heat current balance. The result shows strong evidence that the original implementation of the virial stress heat current formula that involves only pairwise interaction fails to represent the accurate heat current component in the MD simulation, undermining the validity of using the MLIP software in computing κ_*L*_ using the MD approaches. Comparing Figure 2a,b, the introduction of the many-body heat current formula significantly improves the energy balance between heat current within the simulation thermostat system and the intrinsic heat current of the sandwiched region evaluated by MTP. These simulations suggest that the original method underestimates the actual heat current by around 50% and could impact the result of the κ_*L*_ calculation by direct method MD simulations.

**Figure 3:**
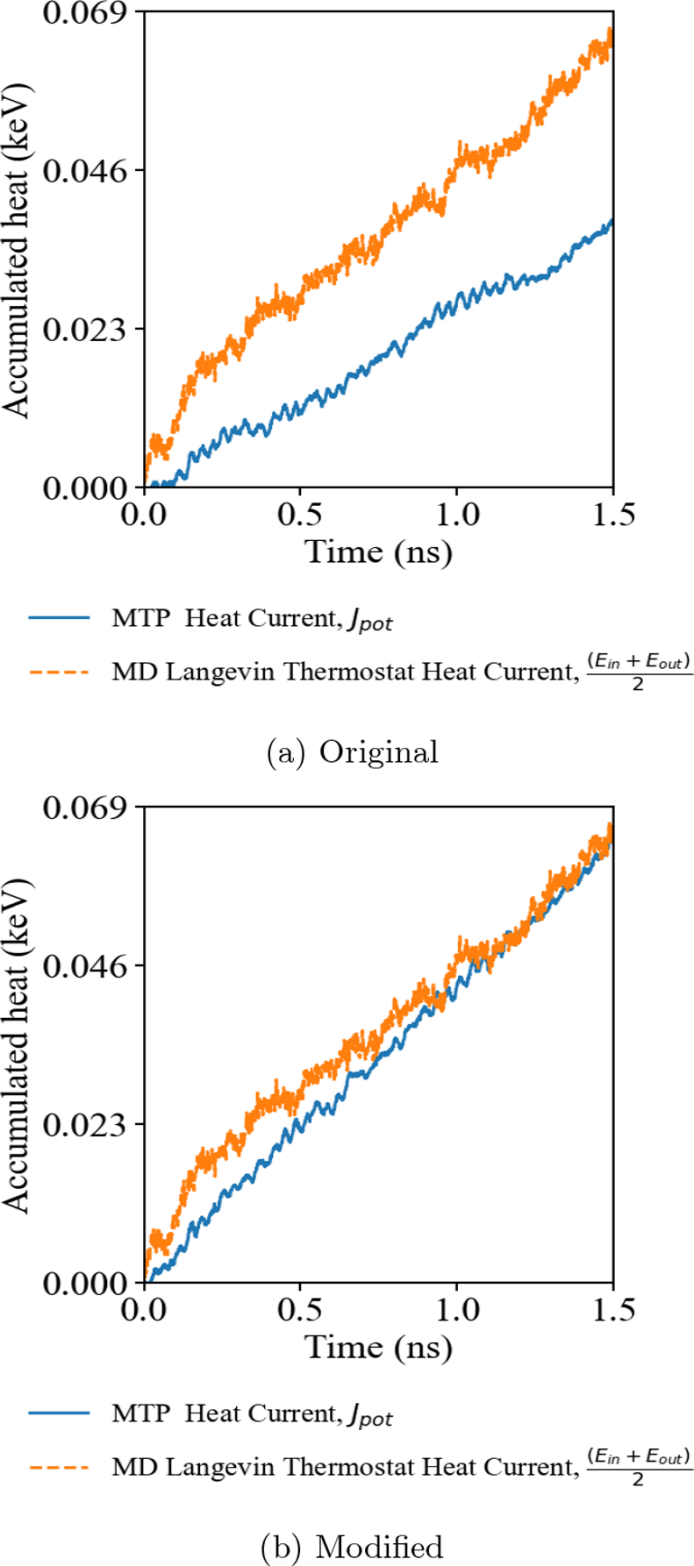
Accumulative heat of *NEMD* simulations of amorphous Sc_0.2_Sb_2_Te_3_ using (a) the original MLIP interface and (b) the modified MLIP interface with the many-body heat current formula correction.

A more complex material with a disordered structure of amorphous Sc_0.2_Sb_2_Te_3_ shows a trend similar to that of PbTe, suggesting that the issue of the MTP original heat current representation is general regardless of the structure complexity of the subject. This ternary phase change material was constructed by the melt and quench of a large unit cell consisting of 180 atoms with a lattice parameter of length up to 18 Å, assuring the randomness of atomic configurations well within the MTP interaction cutoff radius. A similar comparison of the cumulative heat current from the MD simulation heat bath and the evaluated heat current within the simulated domain is shown in Figure 3. The original heat current implementation underestimates the heat current by around 40%, while the many-body heat current correction shows better agreement between the simulation heat bath and the heat current computed by the MTP potential. This result also indicates a significant improvement in the evaluation of heat current using the many-body formula under a disordered and locally more sophisticated atomic neighborhood environment.

**Figure 4:**
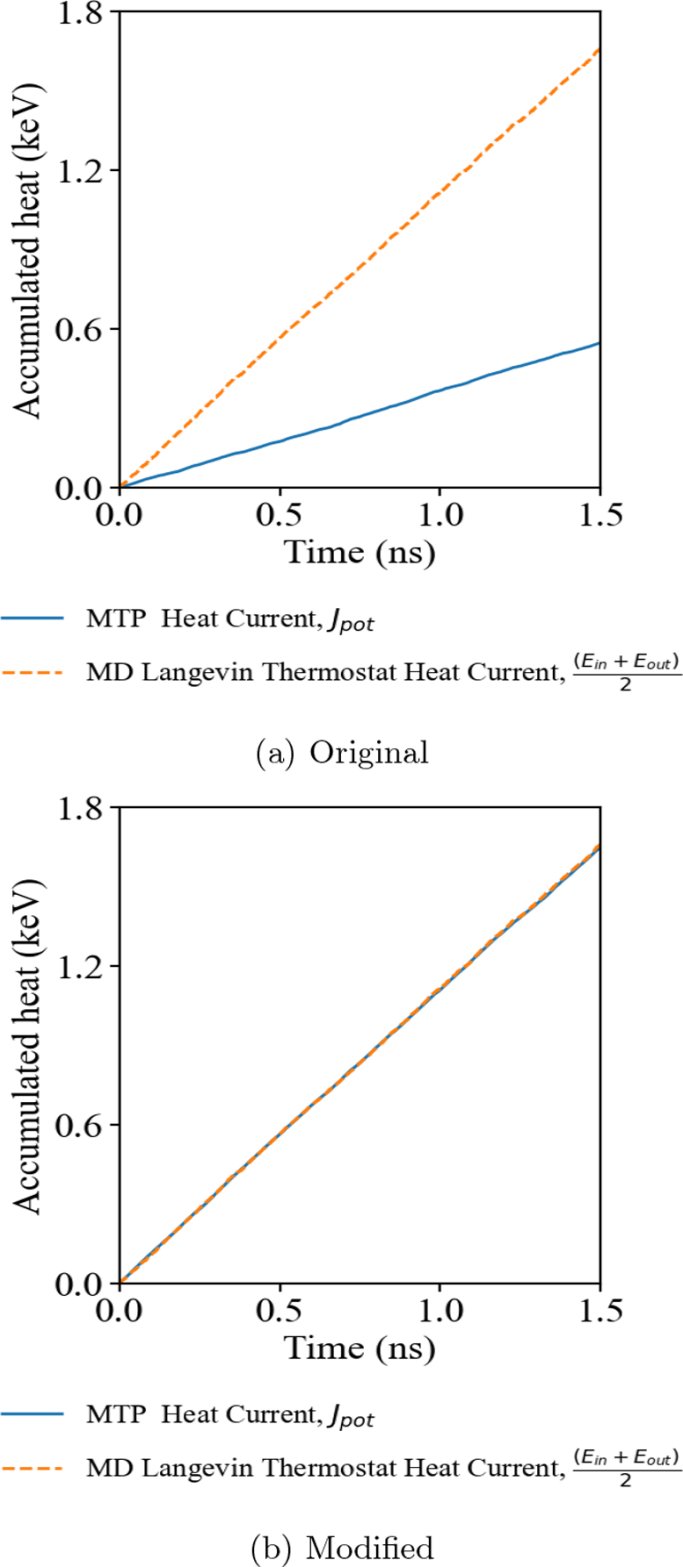
Accumulative heat of *NEMD* simulations of graphene using (a) the original MLIP interface and (b) the modified MLIP interface with the many-body heat current formula correction.

Then, we extend the research to the effect of the many-body heat current correction on 2D material graphene. The cumulative heat current of the nonequilibrium simulations suggests the existence of a similar discrepancy between the average heat current on the ensemble thermostats and the heat current evaluated by the virial stress heat current formulation of the LAMMPS/MLIP interface package across the simulation domain. The original formula leads to the biggest difference among the four testing systems, 75% lower than the MD simulation thermostats. The modified many-body heat current formula shows a significant improvement between the heat baths’ overall heat current and the MTP potential. This result agrees with the recent study of graphene by Dong et al.,^30^ where the MTP potential model was compared with other neural network-based models that had adapted to the many-body heat current interaction description. Figure 4 provides key evidence of the incapability of the original implementation in the MLIP package in determining the heat current in a 2D system.

**Figure 5:**
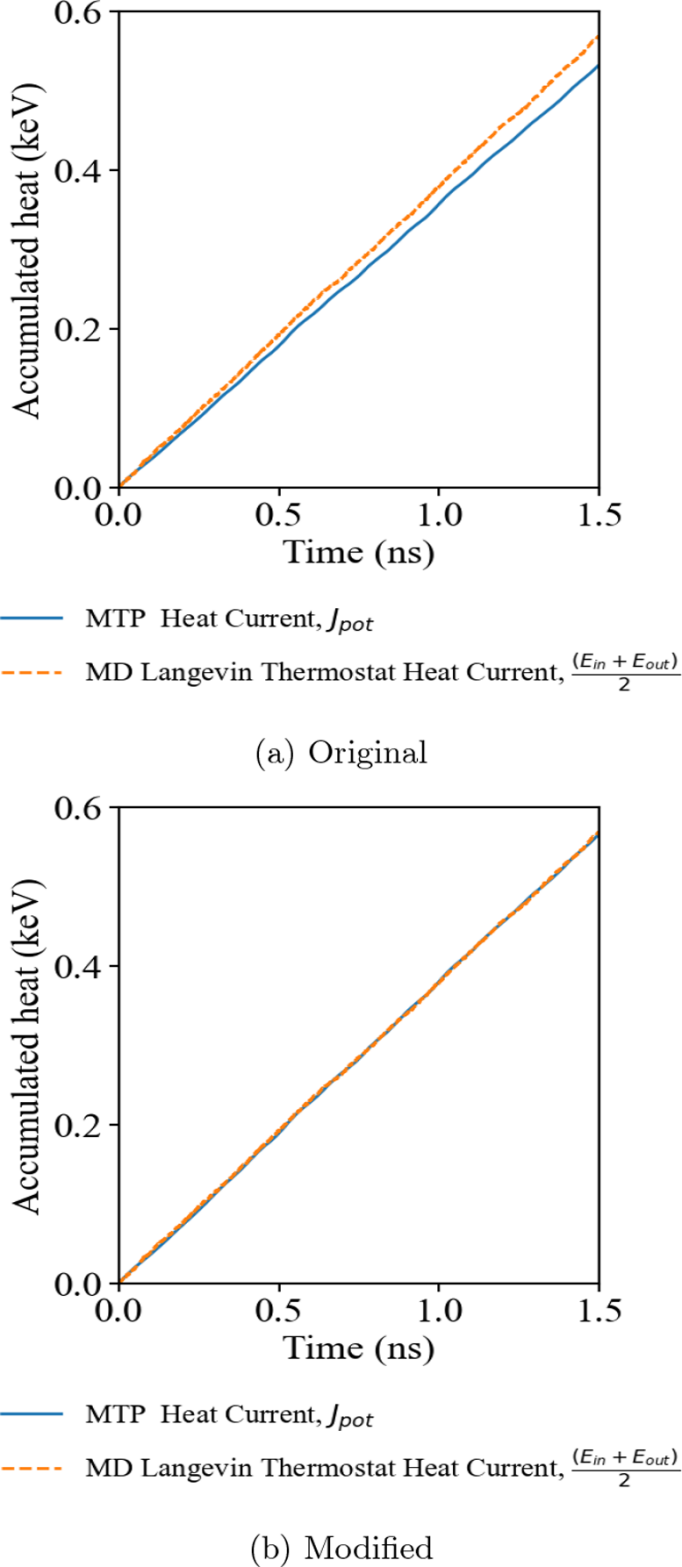
Accumulative heat of *NEMD* simulations of BAs using the (a) original MLIP interface and (b) modified MLIP interface with the many-body heat current formula correction.

Finally, we find in the nonequilibrium simulation that BAs behave differently from the previous three materials. The cumulative heat current of the original implementation in Figure 5 reveals a slight underestimation of the heat current computed by the original heat current expression. The modified many-body heat current results exhibit better agreement between the overall heat current evaluated by the MD simulation thermostat and the MTP computed heat current, while the original pairwise heat current reveals a small deviation in the 1.5 ns time frame. This implies the possibility that the many-body interaction component in the potential component to the heat current is less significant in the case of BAs.

### 3.2 *EMD* Simulation for Thermal Conductivity

**Figure 6:**
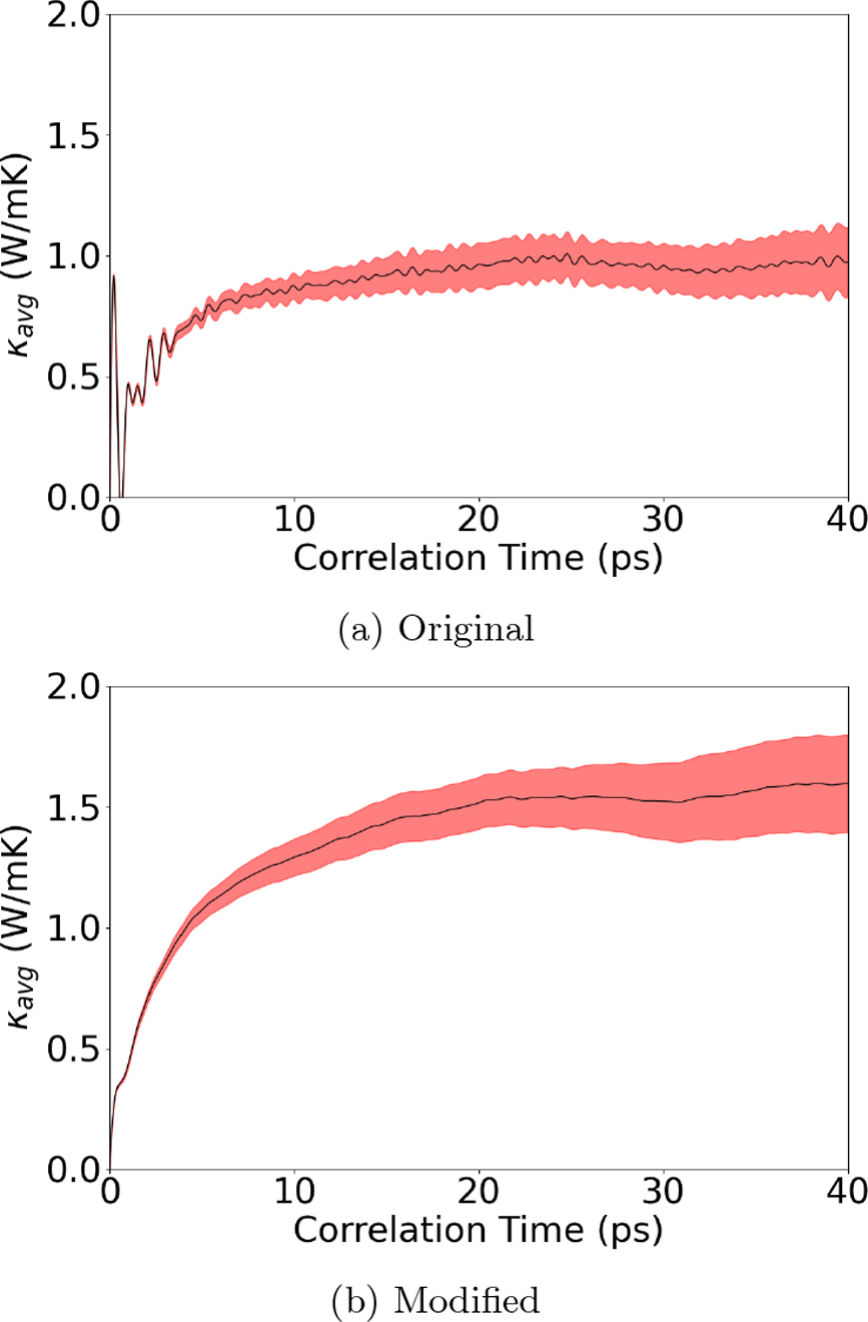
Averaged κ_*L*_ of PbTe at 300 K from 30 independent heat autocorrelation functions against correlation time using the (a) original MLIP interface and (b) modified MLIP interface with the many-body heat current formula correction. Shaded red areas represent the upper and lower bounds within one standard deviation.

We then compare the effect on κ_*L*_ computed based on the Green–Kubo method using the *EMD* approach. Under the same simulation cell size and constraint, PbTe shows a 64% increase in κ_*L*_ from 0.97 to 1.59 W/mK when comparing the original heat current formula and the modified many-body heat current formula. Figure 6 shows the thermal conductivity vs the correlation time of PbTe during *EMD*. The modified formula shows better agreement with the experimental result^33^ of 1.52 W/mK, suggesting that the migration to many-body heat current implementation is a more complete description of the MTP potential.

**Figure 7:**
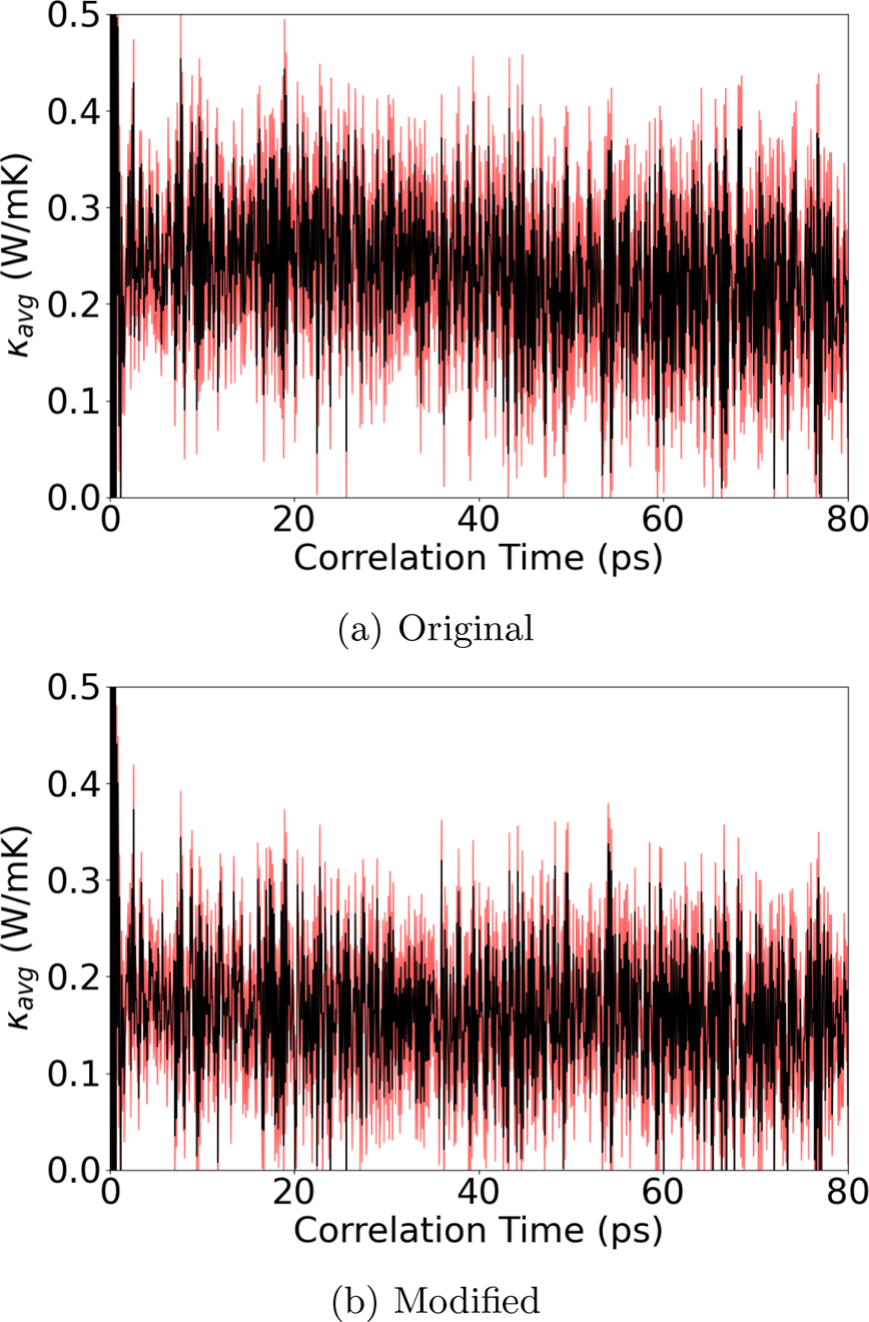
Averaged κ_*L*_ of amorphous Sc_0.2_Sb_2_Te_3_ at 300 K from 30 independent heat autocorrelation functions against correlation time using the (a) original MLIP interface and (b) modified MLIP interface with the many-body heat current formula correction. The shaded red area represents the upper and lower bounds within one standard deviation.

The κ_*L*_ of amorphous Sc_0.2_Sb_2_Te_3_ was then evaluated, which revealed a decrease in κ_*L*_ from 0.21 to 0.15 W/mK. The result shows better agreement with ref (25), in which κ_*L*_ was computed with the Allen–Feldman and the sinusoidal approach-to-equilibrium MD approaches. Figure 7 reveals a noisy, yet distinguishable, change between the modifications. Regarding the previous example of PbTe, one may anticipate a similar increase in κ_*L*_ with a more complete heat current contribution from the many-body interaction component. However, in this case of amorphous Sc_0.2_Sb_2_Te_3_, the impact of including the many-body heat current causes a decrement of 29% in κ_*L*_. The result suggests that the correction to the heat current formula only indicates the potential pitfall of the original heat current value and its autocorrelation and does not guarantee the behavior of its autocorrelation function and its impact on κ_*L*_.

**Figure 8:**
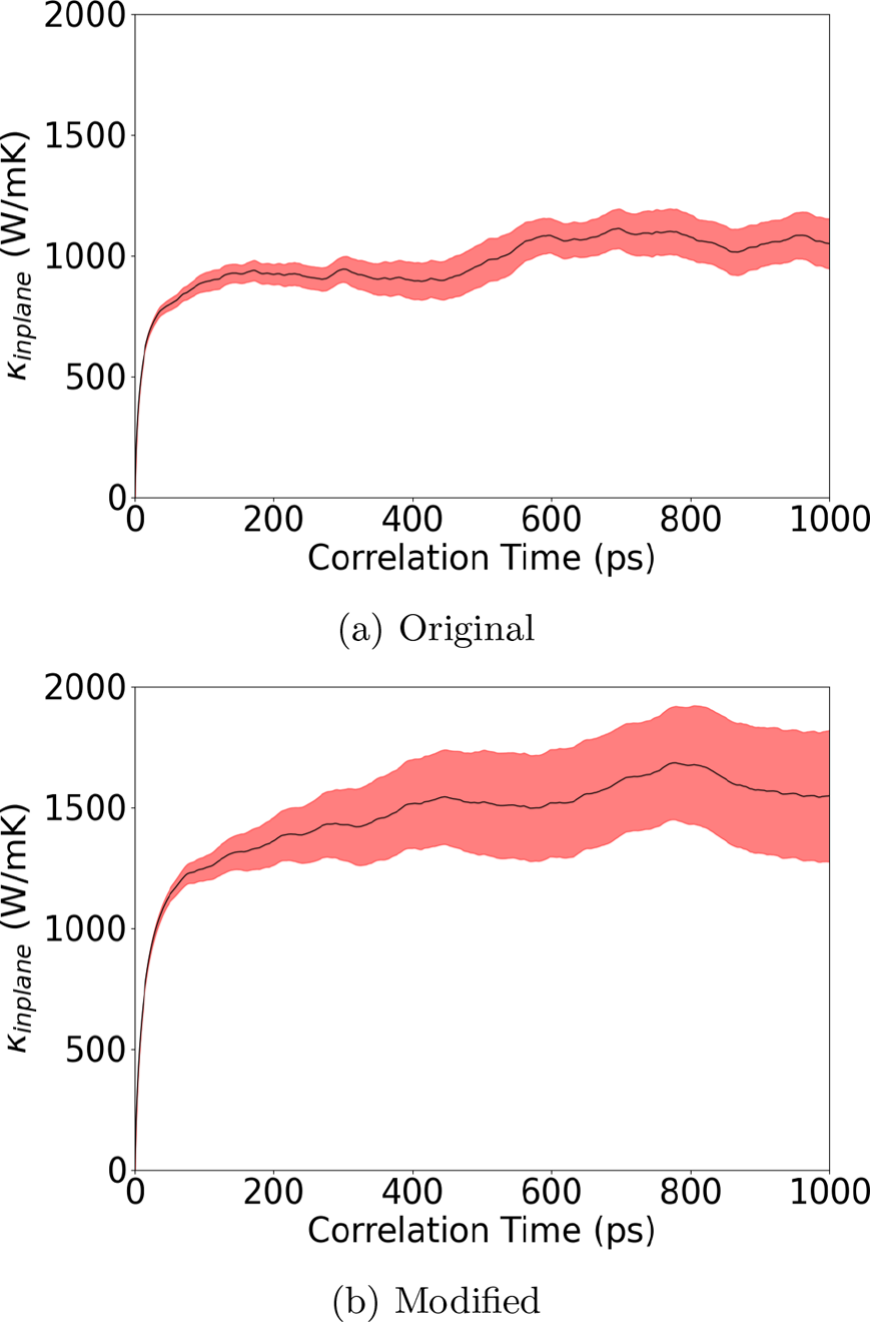
Averaged κ_*L*_ of graphene at 300 K from 30 independent heat autocorrelation functions against correlation time using the (a) original MLIP interface and (b) modified MLIP interface with the many-body heat current formula correction. The shaded red area represents the upper and lower bounds within one standard deviation.

Extending the concept into a 2D material, the Green–Kubo *EMD* result shown in Figure 8 reveals an increment of the heat current autocorrelated thermal conductivity of graphene compared before and after the modification. The κ_*L*_ rises 51% from 1060 to 1605 W/mK, which is considerably smaller than other reported *MD* simulations by Fan,^4^ Zhang,^34^ and Gu,^35^ ranging from around 2300 to 2900 W/mK. The increment of κ_*L*_ suggests that the integration of the many-body contribution of heat flux plays a significant role in the κ_*L*_ of graphene. The discrepancy in our *EMD* result with other literature results can be due to the size effect of our supercell. Similar *MD* results were reported by Pereira and Donadio,^36^ who found that graphene convergence behaves differently when compared to other materials due to failing in sampling the low-frequency acoustic flexural mode at small simulation sizes. A recent work by Fan et al.^37^ further hints at the idea of a lack of flexural mode contribution as they decompose the contribution of in-plane and out-of-plane components to the κ_*L*_ of graphene. It is revealed that graphene thermal conductivity is dominated by an out-of-plane mode up to 60–70%. Increment in κ_*L*_ after the correction implies an underestimation of the in-plane κ_*L*_ contributions in the original MTP formula. Despite the fact that a better agreement with the previous theoretical prediction could be expected by a larger simulation supercell, the computation time is formidable with current computing resources. As we are focusing on the missing accounting effect of the many-body heat current formula to the MTP potential in the existing interface packages, the current result is sufficient to demonstrate the presence of a many-body heat current contribution.

**Figure 9:**
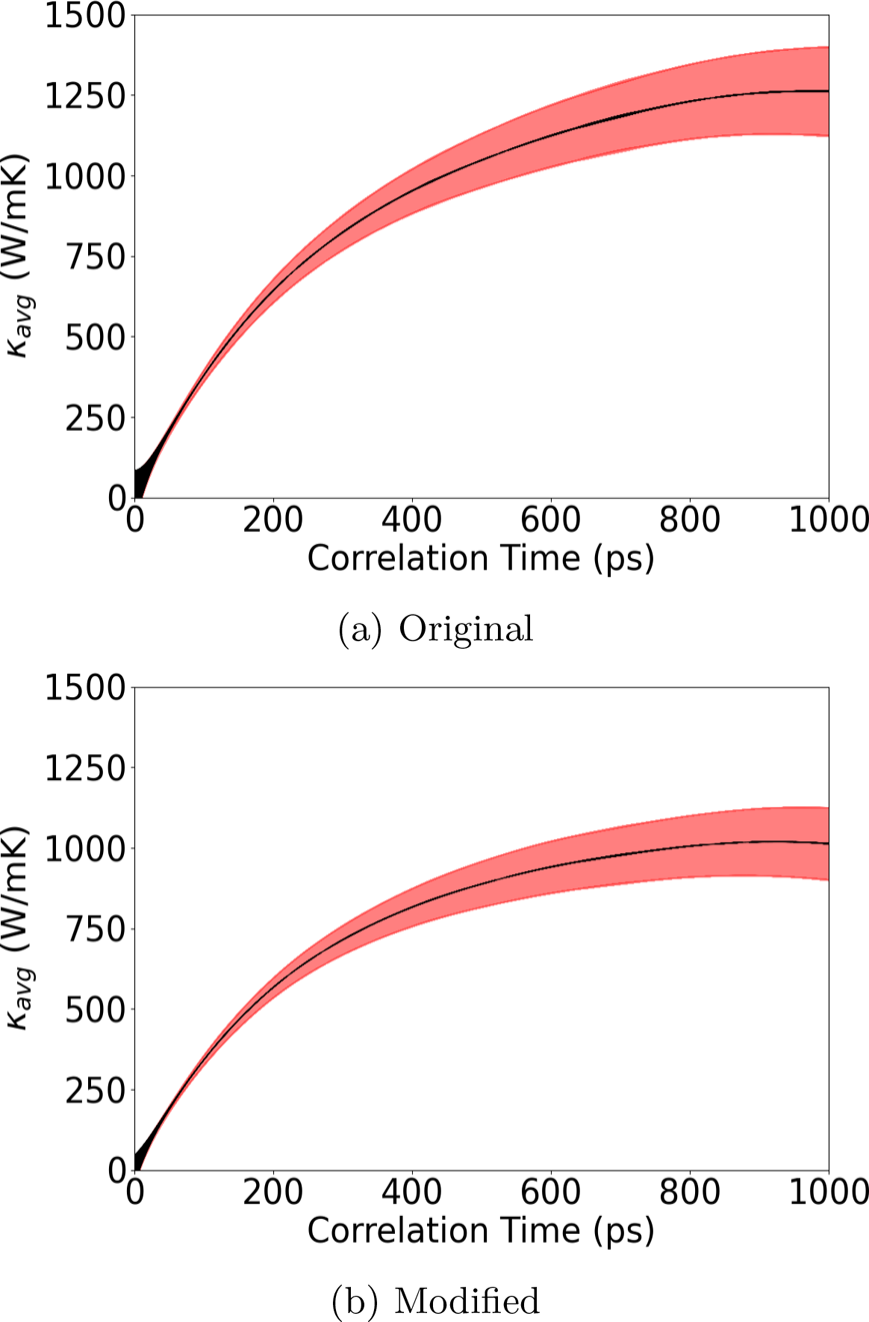
Averaged κ_*L*_ of BAs at 300 K from 30 independent heat autocorrelation functions against correlation time using the (a) original MLIP interface and (b) modified MLIP interface with the many-body heat current formula correction. The shaded red area represents the upper and lower bounds within one standard deviation.

The final example is BAs, in which we also find an interesting trend for the change in κ_*L*_ based on the Green–Kubo method. As shown in Figure 9, a decrease of 19% from 1260 to 1017 W/mK κ_*L*_ is recorded. Compared to the experimental results of Tian et al.,^38^ who reported the κ_*L*_ of BAs at room temperature varies from 450 ± 60 to 1160 ± 130 W/mK across various measurement locations on multiple samples, our result based on MD simulation lies within the range of the measurements. A computation study based on the perturbation theory and phonon Boltzmann’s transport equation (PBTE) reported a κ_*L*_ of around 1400 W/mK incorporating both three- and four-phonon interactions. The difference between the Green–Kubo and *PBTE* methods on κ_*L*_ may be related to the higher-order phonon scattering processes unfolded in *MD* simulations or phonon-boundary scatterings from the size effect. However, as shown in Figure 5, the difference of BAs' overall cumulative heat current between the original and modified formulas is trivial. This indicates that despite an insignificant change in terms of the averaged absolute value of the heat current between the original and the many-body heat current formula, the heat current autocorrelation function can still be altered and distinguishable between the two models.

## 4 Conclusions

In summary, we demonstrated the significance of the many-body interaction when considering the heat current of a model system, and MTP was selected as the workhorse of MD simulation. A modification of the LAMMPS/MLIP interface was implemented based on the generalized many-body heat current formula. Four examples, namely, PbTe, amorphous Sc_0.2_Sb_2_Te_3_, graphene, and BAs, covered an extensive range in terms of the order of magnitude in κ_*L*_ and crystal geometry complexity. All examples except for BAs show a large difference between the MTP evaluated heat current and the actual overall heat current of the NEMD simulation. We further presented that the modification improves the agreement of the MTP calculated heat current and the overall heat current of the MD simulation, suggesting an underestimation of the heat current value without considering the many-body contribution.

The κ_*L*_ comparison of the four examples reveals the significant importance of the corrections. Both low κ_*L*_ materials (PbTe and amorphous Sc_0.2_Sb_2_Te_3_) demonstrated better results in agreement with published experimental and computational data, while graphene and BA results lie within a reasonable range of values. Our calculation suggested that the rectified heat current can impact the computed κ_*L*_ value up to 64%, especially for low κ_*L*_ materials. This work illustrates the significance of adopting the generalized many-body heat current formula and its underlying influence on the thermal conductivity computed based on MD simulation approaches using the MTP heat current operator.

## Data Availability

All original coding and input files for simulation are available at github deposit: https://github.com/taisiuting-alex/Manybody-Heat-Current-MTP-LAMMPS-interface.git.
